# The non-immunosuppressive management of childhood nephrotic syndrome

**DOI:** 10.1007/s00467-015-3241-0

**Published:** 2015-11-10

**Authors:** James McCaffrey, Rachel Lennon, Nicholas J. A. Webb

**Affiliations:** Department of Paediatric Nephrology, Royal Manchester Children’s Hospital, Central Manchester University Hospitals NHS Foundation Trust, Manchester Academic Health Science Centre, Oxford Road, Manchester, M13 9WL UK; Institute of Human Development, Faculty of Medical and Human Sciences, University of Manchester, Manchester, UK

**Keywords:** Idiopathic nephrotic syndrome, Infection, Vaccination, Oedema, Thrombosis, Dyslipidaemia

## Abstract

Idiopathic nephrotic syndrome (INS) is one of the most common renal diseases found in the paediatric population and is associated with significant complications, including infection and thrombosis. A high proportion of children enter sustained remission before adulthood, and therapy must therefore mitigate the childhood complications, while minimising the long-term risk to health. Here we address the main complications of INS and summarise the available evidence and guidance to aid the clinician in determining the appropriate treatment for children with INS under their care. Additionally, we highlight areas where no consensus regarding appropriate management has been reached. In this review, we detail the reasons why routine prophylactic antimicrobial and antithrombotic therapy are not warranted in INS and emphasise the conservative management of oedema. When pharmacological intervention is required for the treatment of oedema, we provide guidance to aid the clinician in determining the appropriate therapy. Additionally, we discuss obesity and growth, fracture risk, dyslipidaemia and thyroid dysfunction associated with INS. Where appropriate, we describe how recent developments in research have identified potential novel therapeutic targets.

## Introduction

Nephrotic syndrome (NS) is one of the most common causes of chronic kidney disease (CKD) in the paediatric population. The cardinal features are oedema, massive proteinuria and hypoalbuminaemia. Children with NS have a decreased quality of life [1], are at risk of a wide range of complications associated with significant morbidity and experience mortality rates of up to 2.7 % [2].

NS can be divided into congenital NS (presentation before 3 months of age), infantile NS (presentation between 3 months to 1 year of age) and idiopathic NS (INS). The largest of these groups is INS, and initial management involves an 8- to 12-week course of oral glucocorticoid (Gc) therapy [3–5]. Approximately 92 % of children with INS will enter remission during this initial treatment and are subsequently classified as having steroid-sensitive nephrotic syndrome (SSNS), while 8 % fail to enter remission and are classified as having steroid-resistant nephrotic syndrome (SRNS) [6].

### Steroid-sensitive nephrotic syndrome

Data from the 1960s reveal that the most common histological diagnosis in SSNS is minimal change disease (MCD), where glomeruli appear normal under light microscopy, but podocyte foot process effacement is detectable by electron microscopy [7]. In current clinical practice, children who enter disease remission during the initial course of Gc therapy do not require a kidney biopsy (unless there are atypical features), as initial clinical response to Gc therapy in children is of greater prognostic significance than histological findings [8, 9]. Children with SSNS generally have a favourable outcome, and the results of older studies suggested they achieve long-term remission during teenage years [10]. However, recent data suggest that over 30 % of SSNS children relapse in adulthood [11, 12].

### Steroid-resistant nephrotic syndrome

The most common histological diagnosis in SRNS is focal segmental glomerulosclerosis, a term which in practice (though not strictly correctly) is used interchangeably with SRNS [7, 13]. Children with SRNS are treated with a range of other (non-Gc) immunomodulatory agents, including mycophenolate mofetil, cyclophosphamide, ciclosporin and rituximab [14]. More than 60 % of children with SRNS who fail to achieve remission with pharmacological intervention will progress to end-stage renal disease [15]. There are also differences in mortality rates according to initial response to Gc therapy: 2.7 % in the INS population overall, 18.5 % in children with SRNS, 6.3 % in children with early relapse following initial remission and 0.4 % in children without early relapse [2].

Over the past 15 years genetic discoveries have vastly improved our understanding of the molecular basis of NS. The PodoNet consortium has recently reported data from a heterogeneous population of 1655 children with SRNS with a median follow-up time of 3.7 years. In this series, SRNS was either congenital (6 %), infantile (7 %), adolescent onset (13 %) or childhood onset (74 %). At the time of last follow-up, 11.7 % of the children required dialysis, 14.2 % had received a kidney transplant and 2.3 % were deceased [16]. Of note, 23.6 % of children from this heterogeneous SRNS cohort had a genetic mutation, in contrast to another series where mutations were found in 66 % of NS cases presenting under the age of 1 year [17]. The most common mutations in childhood NS are found in genes encoding nephrin (*NPHS1*), podocin (*NPHS2*) and Wilms tumour 1 (*WT1*) [13]. The increasing availability of genetic testing in SRNS will help clinicians minimise exposure to immunosuppressive agents since genetic mutations are associated with a poor response to immunosuppression [18].

### INS guidelines

Children with INS are at risk of significant complications, including infection, thromboembolism and dyslipidaemia, and while there are a number of international guidelines for the management of INS [9, 14, 19, 20], few provide guidance regarding the non-immunosuppressive therapy applicable for INS. Here, we review the available literature on non-immunosuppressive treatment options for INS and highlight areas requiring further research.

## Infection

The leading cause of mortality in the childhood INS population is infection, and the annual incidence of invasive bacterial infection is approximately 1–2 % [2, 21]. Accordingly, the parents of any child with INS should be advised to seek medical review if their child develops a fever [9]. The International Study of Kidney Disease in Children (ISKDC) followed almost 389 children with MCD for 5–10 years and reported ten deaths, of which six were due to infection [2]. The importance of infection in INS is further underscored by the observation that the first significant reductions in mortality occurred with the introduction of antimicrobial agents in the 1940s before the widespread use of Gc therapy [22]. Viral infection also poses a significant hazard to children with INS. Varicella zoster virus (VZV), measles or influenza can have devastating consequences in immunosuppressed children with INS, including severe pulmonary disease, multi-organ failure and even death [23, 24]. Potential reasons for the increased rates of infection in NS include disturbances of the complement system [25], defective opsonisation and altered T-cell function [26]. However, strongly persuasive data implicating these possible causes are lacking. Several studies have shown an association between infection and altered serum concentrations of immune-related proteins in children with INS, but none have proven a definitive causal link [25, 27]. For example, hypogammaglobulinaemia is a prominent feature of INS in both relapse and remission. Kemper et al. studied serum immunoglobulins, including immunoglobulin G (IgG) subclasses, in 44 children with SSNS and found that early phases of relapse were characterised by reduced IgG-1, while later phases were characterised by deficiency of IgG-1–3 [28]. Deficiency of IgG-2 persisted for up to 1 year into remission. This altered IgG subclass constitution may result in increased susceptibility to infection. Children with INS are at a higher risk of infection during periods of oedema, but whether the oedema contributes to this increased risk or is simply a marker of severe or unresponsive disease is unclear [21]. A further association has been identified between children with severe hypoalbuminaemia at initial presentation of INS and increased risk of subsequently developing peritonitis [29].

### Sites of infection

Several studies have found common sites of infection in children with INS, however discrepancies in the results do exist. Recent reports are from Southeast Asia, and it is therefore unclear how transferrable these data are to the European or U.S. setting. Furthermore, many studies are uncontrolled in design, creating difficulties for differentiating infections common in the general paediatric population from those specific to the INS population.

A retrospective study from 1968 provided data from 39 episodes of infection in children with INS. Cellulitis and other soft tissue infections were the most common (54 %), followed by peritonitis (21 %) and nonlocalised bacteraemia (21 %) [30]. It is unclear from these data whether the presence of oedema was strongly associated with soft tissue infection. A more recent study from Taiwan reviewed 28 cases of bacterial infection in children with INS presenting over a 10-year period. The authors reported peritonitis in 13 cases, sepsis in six cases, cellulitis in four cases, urinary tract infection (UTI) in four cases and a single case of osteomyelitis [31]. Another Taiwanese study reported pneumonia (49 %) to be the most common infection in children with INS, followed by UTI, sepsis, peritonitis and cellulitis. The authors of this latter study also identified an association between age and site of infection: pneumonia was the most common infection in children with INS aged <10 years, but UTI was more common in children aged >10 years [32]. The importance of respiratory infections in children with INS was also highlighted in a 2003 study from Pakistan which examined 74 episodes of infection and found that acute respiratory infections (29 %) and skin infection (27 %) were the most common, followed by diarrhoea (14 %), UTI (13 %) and peritonitis (11 %). Two children also had pulmonary tuberculosis [33]. An additional study from India involving 154 children found that pulmonary tuberculosis was the second most common site of infection (10 %), preceded by UTI (14 %) [34].

Regarding studies conducted in Western countries, a 1995 study from France reported 32 children with INS requiring admission for intravenous antibiotic therapy. Half of the infections were episodes of peritonitis, with *Streptococcus pneumoniae* accounting for 50 % of the causative agents [35]. Additionally, a 2015 study from Italy reported 218 children with a first presentation of INS. Of these 218 children, 27 (12.4 %) developed infections, with 16 children (7.3 %) having bacterial infections (8 pneumonia, 1 peritonitis, 1 cellulitis and 1 otitis; 5 were not specified), ten having viral infections (enteric or upper respiratory infections and 1 case of primary varicella infection) and one having fungal infection. No data were provided about the causative organism [36]. Further robust data for the Western European population will become available when the ongoing PREDNOS and PREDNOS 2 trials report their findings [37].

### Causative agents

An awareness of common pathogens responsible for infections in children with INS is vital to guide initial antimicrobial therapy. In a U.S. retrospective review focussing on primary peritonitis in INS, *S. pneumoniae* was the major pathogen identified, accounting for 38 % of cases, with Gram-negative organisms cultured from only 3 % of children. An additional 27 % of children had negative culture results but were clinically responsive to penicillin [38]. Another study of peritonitis in children with INS found that *S. pneumoniae* was the most common pathogen identified (50 %), but that *Escherichia coli* accounted for 25 % of cases, and 16 % of cases were culture-negative [39]. A recent prospective study from Turkey monitored 268 children with newly diagnosed INS over a 5-year period and found an incidence of peritonitis of 2.6 %. A microorganism was identified in three out of eight episodes of peritonitis in seven children (*Streptococcus hemolyticus*, *S. pneumoniae* and alpha-hemolytic *Streptococcus*) [40]. *E. coli* was identified as the most common causative agent (61 %) of UTI in children with INS, with non-*E. coli* Gram-negative organisms accounting for 31 % of culture isolates and Gram-positive organisms for 8 % [41]. A study from Taiwan investigating ten episodes of sepsis and eight episodes of peritonitis in 18 children with INS reported that Gram-positive organisms (*n* = 7, with *S. pneumoniae* being the most common) and Gram-negative organisms (*n* = 7) were found in equal numbers. Two of the four cases of *S. pneumoniae* infection in this study were penicillin-resistant [42]. Finally, a case report also documents two infants with NS receiving penicillin chemoprophylaxis who developed penicillin-resistant pneumococcal peritonitis [43]. Overall, there is a preponderance of reports on *S. pneumonia*, providing some justification for the use of penicillin V during relapse of INS.

#### Viral infection

Varicella zoster virus is the most significant viral infection in the INS population and may lead to life-threatening disease in children receiving Gc and other immunosuppressive agents. Dowell et al. performed a retrospective study to determine the risk of varicella infection associated with Gc treatment in children with a range of conditions, including INS, by comparing the proportion of hospitalised cases of severe childhood varicella with concomitant Gc treatment to the corresponding proportion in a sample of children enrolled in a health maintenance organisation [23]. Among these children, 23.6 % had received Gc treatment within 30 days prior to the onset of their rash compared with 0.2 % of controls, giving an odds ratio of 178. The authors report that this could increase the varicella-associated risk of mortality from 3.5 deaths per 100,000 cases to 623 deaths per 100,000 cases [23]. In addition, children receiving immunosuppressive therapy may present with atypical and severe clinical features during episodes of infection with a wide range of viruses [24, 44–46].

Several viral agents, including respiratory syncytial virus, influenza virus, parainfluenza virus, VZV and adenovirus, have been identified as potential triggers of disease relapse [47]. In contrast, exposure to measles virus has been associated with disease remission [48]. A recent paper demonstrated that Epstein–Barr virus (EBV) DNA in whole blood and positive IgM serology are more common in children at the onset of INS than in a non-INS control group [49]. Based on these results, the authors suggest an association between the prevalence of EBV and the onset of INS in children.

### Initial treatment of infection

Although *S. pneumoniae* is the commonest cause of bacterial infection in the child with INS, initial therapy of suspected infection should include broad-spectrum antibiotics until culture and antimicrobial sensitivities are available. The American Academy of Pediatrics (AAP) state that in suspected cases of peritonitis, definitive diagnosis requires the culture of peritoneal fluid [9]. However, in clinical practice, many cases are treated on an empiric basis. Tain et al. recommended that combination therapy be used with children with INS and suspected serious infection, with vancomycin to cover penicillin-resistant *S. pneumoniae* and a third-generation cephalosporin to cover rare Gram-negative microorganisms [42]. Gorensek et al. recommended initiating empiric antimicrobial therapy in suspected INS-related peritonitis with penicillin and either an aminoglycoside or broad-spectrum cephalosporin until culture results are available. If Gram-positive diplococci are observed in the culture fluid, then penicillin alone should be satisfactory [38]. The nephrotoxic effects of aminoglycoside antibiotics should be considered before the appropriate therapy is chosen. Aminoglycosides have a narrow therapeutic window, and therefore diligent monitoring of antibiotic levels in blood, followed by dose-adjustment where necessary, is mandatory for this class of antimicrobials. Table 1 summarises treatment options recommended by the Indian Academy of Pediatrics (IAP) and the Royal Manchester Children’s Hospital (UK) for antimicrobial treatment according to site of infection.

**Table 1:** Management of infections in steroid-sensitive nephrotic syndrome^a^

Infection	Common organisms	Antimicrobial guidance from Indian Academy of Pediatrics	Antimicrobial guidance from Royal Manchester Children’s Hospital, UK
Peritonitis	*Streptococcus pneumoniae, Streptococcus pyogenes, Escherichia coli*	Cefotaxime or ceftriaxone (7–10 days); ampicillin and an aminoglycoside (7–10 days)	Ceftriaxone (avoid aminoglycoside due to potential toxicity if possible).
Pneumonia	*S. pneumonia, Haemophilus influenzae, Staphylococcus aureus*	Oral: amoxicillin, co-amoxiclav, erythromycin Parental: ampicillin and an aminoglycoside; or cefotaxime/ceftriaxone (7–10 days)	Co-amoxiclav or clarithromycin (if penicillin allergy)
Soft tissue/cellulitis	Staphylococci, Group A streptococci, *H. influenzae*	Cloxacillin and ceftriaxone (7–10 days), co-amoxiclav	Flucloxacillin

^a^Adapted from the publication of the Indian Pediatric Nephrology Group et al. [19], used with the permission of *Indian Pediatrics*

### Prevention of infection

#### Chemoprophylaxis against bacterial infection

Currently, it is not standard practice to routinely administer antibiotic prophylaxis in relapse or remission of INS [20]. The AAP states that there are no data supporting the efficacy of prophylactic penicillin in preventing peritonitis in INS [9]. French guidance recommends prescribing antibiotics only if infection is evident [20]. However, some centres, including the Royal Manchester Children’s Hospital (UK) routinely prescribe penicillin prophylaxis (phenoxymethylpenicillin/penicillin V 12.5 mg/kg twice daily) during relapse and stop antibiotic therapy when oedema has abated. The reason for the marked variation in clinical practice may be explained by the lack of data: no randomised controlled trial (RCT) has ever been performed to test the efficacy of antibiotic prophylaxis in INS. However, the existing data bring into question the efficacy of penicillin V in paediatric INS. For example, the literature contains multiple reports of serious infection occurring in children with INS receiving penicillin V prophylaxis [10, 38, 43, 50]. It has been estimated that approximately 110 children with INS would need to be treated for 1 year to prevent one episode of pneumococcal infection [21]. Children with INS are at risk of infection from a range of pathogens, so it is perhaps unsurprising that penicillin monotherapy prophylaxis does not appear to confer significant protection, based on currently available data.

Children with sickle cell disease (SCD) are similarly at increased risk of developing pneumococcal infection, and robust evidence exists that penicillin prophylaxis does reduce infection rates in this population [51]. However, the observation that the risk of pneumococcal infection in INS is lower than that in SCD [51] means that the practicalities of designing a RCT examining the use of chemoprophylaxis in INS represent a considerable hurdle: 3000 children would be needed to determine whether prophylactic penicillin reduces the incidence of invasive pneumococcal disease (with only 80 % power) [21]. The risk of potential drug side-effects and the development of penicillin-resistant organisms must be balanced against the potential benefit of chemoprophylaxis for children with INS. Reassuringly, data from the SCD population suggest that penicillin prophylaxis does not increase the rate of colonisation with resistant strains of *pneumococcus* and that the rate of drug side-effects is low [52]. The criteria for use of prophylactic antibiotics in children with INS have traditionally been an active area of debate. However, the topic has become less relevant following the introduction of universal pneumococcal vaccination.

#### Pneumococcal vaccination

Pneumococcal vaccines are either unconjugated polysaccharide vaccines (e.g. Pneumovax/PPSV23) or protein conjugate vaccines (PCV). Examples of PCV include Prevnar 13, which is a tridecavalent vaccine (i.e. containing 13 serotypes of pneumococcus). In the general population, polysaccharide vaccines have been shown to be ineffective in children under 2 years of age [53], while PCVs are highly effective in preventing invasive pneumococcal disease in children in this population [53, 54]. Universal pneumococcal vaccination is now standard practice in America and most European countries, including the UK [55]. In the UK, PCV is administered to all children at 2, 4 and 12–13 months of age [56]. Children with INS are also offered an additional single dose of pneumococcal polysaccharide vaccine (PPV) when they are ≥2 years of age [56].

The AAP recommends the administration of pneumococcal vaccines to children with INS (if not already immunised) [9]. The IAP recommends two to four doses of the conjugate pneumococcal vaccine for children aged <2 years. For previously unimmunised children aged 2–5 years, a priming dose of the conjugate vaccine is recommended, followed 8 weeks later by a dose of PPSV23. Children older than 5 years require only a single dose of the polysaccharide vaccine. Revaccination after 5 years is considered for children under 10 years old with active INS [19].

To date there has not been a controlled trial with the aim of examining whether pneumococcal vaccination prevents pneumococcal disease specifically in children with INS. However, a Cochrane review examined the efficacy of pneumococcal vaccines in children with SCD, involving five trials with a total of 547 participants [57]. One trial demonstrated that polysaccharide vaccine only slightly reduced the risk of infection in children younger than 3 years of age, while three trials of conjugate vaccines showed increased antibody response compared to control groups of all ages. Unfortunately, no data on clinical outcomes were provided. The authors concluded that conjugate pneumococcal vaccines should be used in individuals with SCD. However, caution should be exercised when extrapolating these findings to the INS population. It is also important to note that not all pneumococcal serotypes are included in the vaccines and that antibody levels may decline during a relapse. Previously vaccinated children may, therefore, develop pneumococcal peritonitis and sepsis [19].

Ideally, immunisation is performed when a child is not currently receiving immunosuppressive therapy. However, data exist suggesting children on Gc therapy can mount a sufficient immune response to vaccination. In order to investigate whether children with INS are capable of adequately responding to PPV, Spika et al. reported on 27 children with SSNS and six children with SRNS with 12 age-matched controls, following vaccination with PPV [58]. Antibody responders were defined as those with at least a twofold increase in antibody titre after vaccination in addition to an antibody concentration of >200 ng of anticapsular pneumococcal antibody nitrogen per millilitre (ngN/mL) after vaccination. The post-vaccination mean total antibody concentration for the control group was 492 ngN/mL, compared to 48 ngN/mL for the SRNS group (not taking Gc), 639 ngN/mL for the SSNS group taking Gc and 937 ngN/mL for the SSNS group not receiving Gc (i.e. higher than controls). Children with SSNS who were not receiving Gc therapy at the time of vaccination had significantly higher antibody titres to five pneumococcal subtypes before vaccination and to seven subtypes after vaccination compared with control subjects (out of 12 subtypes investigated). Fewer children with SSNS receiving Gc achieved total antibody concentrations of >200 ngN/mL against type 19 F compared with children with SSNS not receiving Gc or with control subjects. Overall, these data suggest that PPV vaccination is sufficiently immunogenic in children with SSNS, but not in children with SRNS.

Further data are provided by a French study which demonstrated that children with INS on high-dose Gc therapy respond to the PPSV23 vaccine [59]. Comparison of the serological response in 30 children with INS directly after initiation of 60 mg/m^2^ prednisolone therapy (Group 1) with the response in 13 children who received the vaccine while in remission (Group 2) revealed that both groups demonstrated an approximate tenfold increase in pneumococcal antibody levels within 1 month, but that this had fallen to approximately three- to fourfold above baseline by 1 year (no difference between Group 1 and Group 2).

Although decay of pneumococcal antibody levels post-vaccination is well recognised in healthy children, this decay seems to be accelerated in children with INS and other CKD [56]. Spika et al. divided a group of 25 children with SSNS into a subgroup of non-relapsers and those who had at least one relapse and observed that the relapsing group had a more rapid decline in total anticapsular antibody per month than the non-relapsing group [60]. In a much more recent study, Guven et al. examined IgG antibody levels against specific pneumococcal antigens before and after PPV administration in nine children with SSNS during remission while off Gc therapy. Although a good initial antibody response was found, by 6 months post-vaccination, IgG levels had begun to fall in six of the nine children, and by the 36th month post-vaccination only two patients still had high IgG concentrations [61]. Given these data, it is unsurprising that multiple cases of pneumococcal disease after vaccination have been reported in the NS literature [38, 62, 63]. An altered IgG-subclass distribution in children with INS may also contribute to a sub-optimal response to pneumococcal vaccination.

#### Influenza vaccination

Children with CKD have a higher risk of influenza virus infection than healthy subjects [56]. Immunosuppressed children, including those with INS, occasionally experience severe complications following influenza A infection, but the disease is mostly mild and self-limiting. An adult study of 25 immunocompromised patients (bone marrow and renal transplant recipients, and those with haematological malignancies) who developed influenza A infection reported that only two patients developed serious infections [64]. A study involving 19 children with INS and ten healthy controls showed that children with INS mount an adequate response to influenza vaccine, with the authors reporting no difference in mean concentration of specific IgG antibodies to influenza A or protective antibody titres (≥1:40) between the groups at 1 month post-vaccination. These authors followed up eight of the children for 6 months post-vaccination (none of the controls) and found that these eight children still had protective antibody titres at the 6 month follow-up visit, and that there was still a statistically significant difference in the mean concentration of specific IgG antibodies compared to the pre-vaccination concentration [65].

The AAP recommends annual seasonal administration of influenza vaccine to children with INS [9]. Currently there are two influenza vaccines available: (1) an inactivated intramuscular vaccine and (2) a live attenuated intranasal vaccine. The live intranasal formulation is contraindicated in children on high-dose Gc therapy. Current UK guidance suggests that children receiving either of the vaccines for the first time should receive a second dose 1 month after administration of the first [56].

#### Varicella zoster vaccination

Infection with VZV is associated with significant morbidity and mortality in children receiving immunosuppressive therapy, including those with INS [23, 66]. The clinician should also be aware that the diagnosis of varicella may be obscured by atypical or absent skin lesions in the immunocompromised child [67]. The AAP recommends varicella vaccination for non-immune children with INS, post-exposure immunoglobulin for non-immune immunocompromised children and the consideration of intravenous acyclovir for immunocompromised children at the onset of varicella skin lesions [9].

Data from 1997 showed that the varicella vaccine is safe in children with SSNS, but the authors found that a second vaccine dose was necessary before seroconversion was achieved in four of seven children and therefore recommended a two-dose vaccine schedule [68]. A U.S. study found that a two-dose varicella vaccine regimen in 29 children (13 of whom were receiving alternate-day Gc) produced protective levels against VZV and that these levels were maintained 2 years post-vaccination (children were excluded if they had received cytotoxic immunosuppression in the previous 3 months, or daily Gc therapy, ciclosporin or tacrolimus within 1 month of enrolment) [69]. No adverse effects were associated with vaccination. A study from Turkey examined single-dose VZV vaccination in 20 children with SSNS and 22 healthy controls (children with SSNS were either in remission or had already stopped Gc therapy for at least 6 weeks prior to vaccination) [70]. Among the 20 children with INS, 85 % seroconverted 8 weeks after vaccination compared to 86 % in the control group. Two years after vaccination, VZV antibodies were still detectable in 70 % of children with INS but in only 59 % of healthy controls. These data support the use of the standard two-dose VZV vaccination schedule in this population. Current UK Department of Health guidance recommends that VZV vaccine should be given at least 3 months after high-dose Gc therapy has been discontinued (prednisolone 2 mg/kg/day for at least 1 week, or 1 mg/kg/day for 1 month). Advice from an immunologist should be obtained regarding the use of the varicella vaccine in children receiving other types of immunosuppressive drugs (e.g. cyclophosphamide) alone or in combination with lower-dose Gc therapy (especially during the 6 months following discontinuation of immunosuppressive therapy) [56]. Finally, some concern has been raised about the possibility of varicella vaccination causing INS relapses, but definitive data supporting this hypothesis are currently lacking. One study reported that among the 20 children with INS, one had a relapse 3 weeks after varicella vaccination [70]; another study reported relapses in three of seven children with INS [68]. However, in both studies, the pattern of relapses in affected children was similar pre- and post-vaccination, and no causal link could be made.

#### Management of exposure to VZV in children with INS

When a child with INS is exposed to VZV, knowledge of immunity status is vital to guide management. Following a study which demonstrated that self- or parent-reported history of previous varicella infection is not highly predictive of seropositivity among cohorts of unvaccinated persons born since 1994 [71], the U.S. Advisory Committee on Immunization Practices (ACIP) has reviewed its criteria for patient groups considered to have been exposed to VZV [72]. Evidence of immunity to varicella is now limited to the following: (1) documentation of age-appropriate vaccination with a varicella vaccine; (2) laboratory evidence of immunity; (3) birth in the USA before 1980; (4) diagnosis or verification of a history of varicella disease or herpes zoster by a healthcare provider. When a VZV naïve (according to the above criteria) immunosuppressed child is exposed to VZV, passive immunoprophylaxis and/or aciclovir should be considered. Significant exposure to varicella may be defined as “residing in the same household, face-to-face indoor play for more than 1 h and hospital contact with an infectious individual (same 2- to 4-bed room or in adjacent beds or a visit in room by contagious person for more than 1 h)” [73]. The two choices for passive immunisation against varicella infection are varicella zoster immunoglobulin (VZIG) and a purified human varicella zoster immune globulin prepared from plasma containing high levels of anti-varicella IgG (VariZIG).

Children should receive passive varicella immunisation as soon as possible after exposure [74], but there are some data suggesting that the incidence of varicella is comparable among children who receive VZIG within 4 days of exposure and those who receive it >4 days (up to 10 days) after exposure and that attenuation of the disease might be achieved with administration of VZIG up to 10 days after exposure [74]. VZIG is given by intramuscular (IM) injection, but children with bleeding disorders who cannot receive IM doses should be given intravenous normal immunoglobulin. Studies from the 1970s demonstrated that VZIG favourably modified disease severity and reduced susceptibility to VZV in immunocompromised children following household exposure [75, 76]. A further study found that VZIG provided protection against severe infection in immunocompromised children following varicella exposure although 60 % of 81 recipients of VZIG still experienced clinical VZV infection [77].

Evidence supporting the use of aciclovir following exposure to varicella comes from a 1993 Japanese study [78]. In this study, oral aciclovir was given to 25 exposed infants and children for 7 days, beginning 7–9 days post-exposure. At the end of the study period, clinical features of the 25 exposed infants who received aciclovir were compared with 25 age-matched exposed patients who did not receive aciclovir. Only 16 % of the subjects treated with aciclovir developed the disease, while all 25 individuals in the control group developed VZV infection. The incidence of fever and the severity of skin rashes were significantly lower in the subjects receiving acyclovir than in the control group. A separate study examined the efficacy of post-exposure aciclovir prophylaxis in children receiving Gc treatment post-renal transplant or for INS [79]. Group 1 received oral aciclovir 7 days after exposure for 7 days. Both groups additionally received VZIG. No child in Group 1 (*n* = 8) developed chickenpox compared to 25 % of the children in Group 2. However, the study size was too small for the authors to draw any definitive conclusions. The IAP recommends that children who develop VZV infection should receive intravenous aciclovir (1500 mg/m^2^/day in 3 divided doses) or oral aciclovir (80 mg/kg/day in 4 divided doses) for 7–10 days. The guidelines also recommend that the dose of prednisolone should be tapered to 0.5 mg/kg/day or lower during the infection [19].

#### Measles infection

Measles can be a devastating illness in immunosuppressed children with INS [24]. It is important to document the measles status for all children with INS (measles immunisation is a routine part of childhood vaccination in the UK, Europe and USA). Measles-specific immunoglobulin therapy should be considered if a non-immune children with INS comes into contact with measles (aciclovir is not effective).

## Adrenal insufficiency

During periods of infection, the clinician should pay careful attention to the dose of Gc prescribed: infection is a significant stressor and failure to increase Gc supplementation during these periods is a significant cause of morbidity and mortality due to acute adrenal insufficiency [80]. In a study evaluating rates of adrenal suppression in ten children with acute lymphoblastic leukaemia receiving a 4-week course of Gc therapy (oral dexamethasone 6 mg/m^2^ per day/approximately 0.2 mg/kg per day, divided into two daily doses), all ten children had normal adrenal function before commencing Gc treatment and an insufficient adrenal response 24 h after completing therapy (evaluated using the short Synacthen test). Of these ten children, three did not regain normal adrenal function until 8 weeks after discontinuation of Gc therapy [81]. In another study involving children with INS, those receiving long-term alternate-day Gc treatment (mean prednisolone dose 0.40 mg/kg) were found to be at risk of developing adrenal suppression (again evaluated using a short Synacthen test), and those with a suboptimal cortisol response were at greater risk of disease relapse [82]. Children with INS on prolonged prednisolone also remain at risk of adrenal insufficiency until at least 9 months after discontinuation of Gc therapy [83].

Controversy exists regarding the level of symptomatic infection that requires increases in Gc dose. Guidance from the Pediatric Endocrine Society suggests uncomplicated viral illness and upper respiratory tract infections with sore throat, rhinorrhoea, otitis media and/or low-grade fever may not require treatment with a stress-dose steroid regimen if the child otherwise appears well. Conversely, illness accompanied by fever of ≥38 °C should be accompanied by increased Gc doses [80]. The degree to which doses should be increased is also under debate, with recommendations varying between two- and tenfold the ‘maintenance rate’ [80]. If parenteral Gc is required due to inability to tolerate oral maintenance therapy, hydrocortisone is the preferred agent due to its additional mineralocorticoid activity [80]. Recommendations specific to INS are extremely limited, but the IAP recommends that children who have received high-dose Gc therapy within the last 2 years require supplemental doses of Gc during periods of serious infection [19]. The guidance does not formally define ‘high-dose steroids’ or provide guidance as to what steroid supplementation should entail. However, the UK Department of Health defines ‘high-dose’ Gc therapy as prednisolone 2 mg/kg/day for at least 1 week or, alternatively, 1 mg/kg/day for 1 month [56].

## Thromboembolic disease

Thromboembolic disease (TED) is the second leading cause of mortality in the nephrotic child [2, 84]. A large review using data from the U.S. National Discharge Survey found that in the 18- to 39-year-old group, the relative risk of deep vein thrombosis in INS patients, compared to patients without INS, was 6.81 (no data was provided for the paediatric population) [85]. INS-associated thromboembolism is much more common in adults (20–52 %) [86–89] than in children (childhood prevalence of 2–13 %) [31, 90–99].

A study involving 326 children with INS and a median follow-up time of 3.7 years reported that 9.2 % of the patient cohort experienced at least one episode of TED. Deep venous thrombosis was the most common TED event (76 %) and was frequently associated with the use of a central venous catheter (45 % of deep venous thrombosis events). Unfortunately, it is unclear whether the presence of a central venous catheter was simply a marker of disease severity, or whether the presence of the catheter itself conferred increased risk for TED [98]. These results were similar to those reported by Lilova et al., who found that TED in children with INS was predominantly venous (81 % vs. 19 % arterial), with the most commonly affected vessels being the deep leg veins, followed by the inferior vena cava [96]. Although arterial thrombosis occurs less frequently than venous thrombosis in INS, it may cause devastating cerebral, abdominal, peripheral and renal infarction. Suri et al. reported on seven children with INS who experienced central nervous arterial infarcts and two children with thrombosis of peripheral arteries [100]. The children with intracranial arterial thrombosis presented with either an acute stoke/hemiparesis-like event or seizures, while the remaining two children experienced involvement of either the posterior tibial or brachial artery, leading to progressive gangrene. TED in children with INS generally occurs early in the disease course, with a median time from diagnosis to the first TED event of 71 days [98]. Clinicians should be alert to the possibility of TED soon after the initial diagnosis of INS.

### Pathophysiology

The underlying reasons for high TED rates in INS are poorly understood, but they are likely to be multifactorial [99]. General background risk factors, such as the presence of known thrombophilic genetic mutations (e.g. congenital antithrombin deficiency) or the placement of central venous catheters, may place the child with INS at higher risk for TED [101]. Sahin et al. provide adult data suggesting that coexistence of inherited thrombophilia in NS may increase the risk for TED. In this study of 51 newly diagnosed INS patients, six (11.8 %) had TED at time of diagnosis (4 symptomatic, 2 subclinical), all of whom carried mutations for either the pro-thrombotic Factor V Leiden, prothrombin or methylenetetrahydrofolate gene [102]. The observation that the fibrin clot formed by nephrotic serum has an altered molecular structure and is more resistant to fibrinolysis compared to fibrin clots from healthy controls is another plausible explanation of the thrombotic tendency in children with INS [103].

Another frequently cited reason for increased TED rates in children with INS is urinary loss of key regulators of haemostasis during relapse. Unfortunately, data directly examining the link between such derangement and TED risk are scarce [99]. However, it is clear that the children with INS do have an altered haemostatic milieu. For example, plasma concentrations of higher molecular weight procoagulant proteins, such as factor V and factor VIII, are elevated in the nephrotic state [94]. Additionally, urinary loss of the lower molecular weight (anticoagulant) protein antithrombin (AT) may shift children with INS to a prothrombotic state [94]. A raised platelet count is an extremely common finding in INS [99]; however, it is unclear whether the reactive thrombocytosis seen in INS actually increases the risk for TED [104]. Eneman et al. recently demonstrated that urinary losses and resultant plasma deficiency of an inhibitor of megakaryopoiesis and platelet aggregability, pituitary adenylate cyclase-activating polypeptide (PACAP), may play a role in the pathogenesis of platelet dysfunction in INS [105].

Ideally, a biomarker for predicting TED risk for children with INS would be available to clinicians. Although this remains a future goal, a recent study from China has shown that adult INS patients with TED had higher levels of two markers of endothelial injury, namely, circulating endothelial cells and von Willebrand factor, compared to adult patients without TED disease and healthy controls [106].

### Diagnosis and investigations

Signs and symptoms of TED include a painful, swollen extremity in cases of venous thrombosis and/or respiratory compromise in cases of pulmonary embolism (PE) [107]. In addition, headache or visual disturbance may reflect an underlying sagittal sinus thrombosis. Any suspicion of clinical TED should prompt urgent imaging to confirm the diagnosis. Computerised tomography pulmonary angiography (CTPA) is the modality of choice for patients with clinically suspected PE [108]. Doppler compression ultrasonography is used as first-line imaging for suspected venous thrombosis, but if images are suboptimal, magnetic resonance venography can be utilised [107].

Data are limited to help decide whether children with INS should be routinely and regularly imaged (irrespective of clinical signs/symptoms). Hoyer et al. imaged 26 children with SSNS in the acute phase of a relapse but without symptoms of TED using combined scintigraphic pulmonary ventilation and perfusion scans and found signs consistent with PE in 28 % of children and residual changes consistent with previous PE in 39 % [92]. CTPA is now considered superior to ventilation–perfusion scans for the diagnosis of PE due to similar sensitivity but improved specificity [108]. A more recent CTPA study found similarly high rates of subclinical PE in NS children (28 %) [109]. In summary, a significant proportion of TED in INS is subclinical, and the medical team should always be alert to the possibility of TED-related complications in children with INS.

Standard laboratory tests in the child with proven or suspected TED should include platelet count, prothrombin time, activated partial thromboplastin time, thrombin time, fibrinogen and quantitative D-dimers. Measurement of AT may be warranted in severe cases or in children not responding adequately to anticoagulant therapy (see below) [107].

Few studies have been conducted to determine the contribution of genetic prothombotic defects to the overall risk for TED in INS [100]. Fabri et al. tested 53 children with INS for thrombophilic genetic defects and reported that TED events occurred in six of these 53 children. An inherited risk factor was identified in seven children, none of whom experienced a TED event [110]. American College of Chest Physicians Evidence-Based Clinical Practice (ACCP) Guidelines for Children do not include the presence of inherited thrombophilia to guide the duration of antithrombotic therapy, and there are no evidence-based guidelines for thromboprophylaxis in children with thrombophilia. Therefore, genetic testing is not routinely endorsed outside of a research setting [107, 111, 112]. The AAP recommends the evaluation of children with INS and TED for an underlying hypercoagulopathy but does not specify whether this should involve genetic testing [9]. Although robust evidence supporting the introduction of routine screening for genetic causes of thrombophilia in INS is lacking [113], some centres support the use of genetic testing as these children may benefit from close follow-up and longer duration of treatment should a TED event occur [110, 114].

### Primary prevention

The decision to begin prophylactic therapy for any medical condition rests on the balance between potential side-effects of the prophylactic therapeutic strategy and the benefit of disease prevention. Clinicians cannot make an appropriately informed decision regarding the use of prophylaxis to prevent INS-related TED because no large, randomised trials to determine the safety and efficacy of this approach have been performed to date. However, it is unlikely that pharmacoprophylaxis for all children with INS will be a desirable strategy. It has been estimated that if pharmacoprophylaxis were to be universally applied, 75–97 % of children with INS would receive unnecessary anticoagulation and potentially suffer subsequent anticoagulation-related complications, such as bleeding [107]. Although some authors have proposed the use of prophylactic aspirin in this patient group [115], clinicians should be aware of the potential risk of acute kidney injury associated with nonsteroidal anti-inflammatory medications [116]. The IAP states that there is no role for prophylactic treatment with anticoagulants in children with INS (even during periods of hypoalbuminaemia and oedema) [19]. Guidelines from France acknowledge that no consensus regarding TED primary prophylaxis exists. However, they suggest that children with a more severe disease phenotype (albumin <20 g/L; fibrinogen >6 g/L; antithrombin III <70 %; D-dimer >1000 ng/mL) may be considered for prophylaxis with aspirin or low molecular weight heparin (LMWH). Finally, a retrospective cohort study has suggested that statin therapy may lower the risk for venous thromboembolism (VTE) in adults with INS, but more robust data are needed [117]. Recommendations to guide use of primary prevention strategies for INS await definitive trials. The AAP recommends that during periods of disease activity and increased thromboembolic risk, children should be encouraged to continue physical activity and avoid prolonged bed rest [9].

### Treatment

Once TED has developed, prompt anticoagulation should be initiated. The ACCP guidelines state that a child with a first VTE should receive an initial minimum 3- to 6-month course of anticoagulation [111]. In children, this is typically initiated with LMWH. Published weight- and age-based nomograms are available for LMWH dosing in children [111, 118]. Warfarin treatment may begin concurrently for longer term anticoagulation, and as warfarin is cleared hepatically, renal dose-adjustments are generally not required. In contrast, many heparin compounds are cleared by renal excretion and, therefore, special attention should be paid to any sign of renal dysfunction and the dose altered accordingly if necessary [99].

The mechanism of action of heparins is dependent on AT and, very rarely, AT supplementation may be necessary to achieve an adequate anticoagulant effect. Some isolated reports exist of clinicians administering fresh frozen plasma to correct for the expected AT deficiency, irrespective of actual AT levels, but this is not routine practice [96]. No published INS-specific optimal AT target levels exist, although a small case series shows the effective use of recombinant AT in adults with congenital AT during the peri-operative period [119]. In children with INS, titrating LMWH therapy to anti-Xa levels can result in more effective anticoagulation [120]. As yet no data regarding the use of new oral anticoagulants exist specifically for children with INS, although this is a promising possibility for future management [121].

Thrombolytic therapy should be considered for life-, organ-, or limb-threatening TED [111, 122, 123]. One example of organ-threatening TED is bilateral renal venous thrombosis. Consideration of the associated bleeding risk should be made before therapy with a thrombolytic agent is commenced. This is particularly important for premature neonates in whom TED is more common. A recent trial involving 30 neonates treated with LMWH found a poor therapeutic response to recommended doses based on measured anti-Xa levels, and the authors suggest higher initial doses may be required to achieve therapeutic anticoagulation [124]. No robust data on the safety and efficacy of thrombolytic therapy in children with INS exist, but effective and safe use of streptokinase [96, 125] and tissue plasminogen activator [126] for severe TED has been documented in children with INS.

## Oedema

Oedema is a cardinal feature of INS and may lead to respiratory and functional constraints (such as impaired vision because of swollen eye lids and reduced mobility because of severe lower limb oedema) [9, 127]. Traditional teaching suggests that oedema is associated with intravascular hypovolaemia, which may lead to acute, pre-renal, renal failure. Signs and symptoms associated with this oedematous hypovolaemic state include tachycardia, abdominal pain, cool peripheries, oliguria and hypotension [128]. However, children with oedema associated with INS do not universally experience intravascular volume depletion; in fact, some studies suggest that children with INS may have normal or even increased intravascular pressure [129, 130].

Two competing theories to explain INS-associated oedema have been proposed: the ‘underfill’ [131] hypothesis and the ‘overfill’ hypothesis [132–134]. The underfill hypothesis suggests that INS-associated proteinuria and associated hypoproteinaemia leads to reduced plasma colloid osmotic pressure, hypovolaemia and stimulation of the renin–angiotensin–aldosterone system, resulting in salt and water retention. Supportive data do exist which suggest that reduced intravascular volume, reduced glomerular filtration rate (GFR) and increased renin and aldosterone levels are features of INS [135].

Observations that plasma renin activity and reduced intravascular pressure are not universal characteristics of INS led to the overfill theory being proposed, with INS-associated oedema being primarily a defect in sodium excretion [133]. The overfill theory has several critics and is not universally accepted [26, 136]. However, it is clear that the standard (‘underfill’) doctrine of hypovolaemia and salt retention resulting from hypoproteinaemia is an oversimplification. Vande Walle et al. demonstrated that children with significant proteinuria can present both with, and without, hypovolaemic symptoms and laboratory signs, despite equally severe hypoproteinaemia and that sodium retention precedes hypoproteinaemia [128].

### Management

Mild oedema can often be managed conservatively with fluid restriction to two-thirds of maintenance and dietary sodium restriction to <2 mEq/kg/day [137]. Strict monitoring of body weight to assess the efficacy of these interventions is required. However, severe, symptomatic oedema may require pharmacological management, including loop diuretics, thiazide diuretic and/or 20–25 % albumin solution. When the use of a pharmacological agent is considered, it is necessary to differentiate ‘underfill’ (i.e. intravascular volume depletion) and ‘overfill’ (i.e. primary renal sodium retention) oedema [138]. Clinically it is often difficult to differentiate between these two states. However, Schrier et al. provide a number of useful indicators which would suggest an underfilled patient: (1) the presence of postural hypotension, (2) a history of MCD, (3) a serum albumin level of <20 g/L and (4) an estimated GFR (eGFR) of >75 % of normal. In contrast, hypertension, a serum albumin level of >20 g/L and an eGFR of <50 % of normal would suggest an overfilled patient [136].

Urinary indices are also useful in determining the volume status in children with nephrotic oedema. A study from 2009 examined the use of fractional excretion of sodium (FeNa) in 30 children with INS admitted to hospital with oedema (patients on diuretics or receiving immunosuppressive therapy on admission were excluded). The authors reported a FeNa value of 0.2 % could distinguish patients with intravascular volume contraction (FeNa <0.2 %) from those with intravascular volume expansion (FeNa >0.2 %). In this study, ten of the 11 patients with FeNa values of >0.2 % were successfully treated with diuretic therapy alone (intravenous furosemide and oral spironolactone) [137].

Some centres also advocate the use of the quotient of urine potassium and urine sodium + potassium [U_K_/(U_Na+K_) × 100 %], which is an indicator for sodium/ potassium exchange at the distal nephron [139]. Vande Walle et al. suggest that if a low FeNa and a U_k_/(U_Na+K_) ratio of >60 % are measured, an albumin infusion can safely be administered if clinically required. In contrast, patients with a normal FeNa and a U_k_/(U_Na+K_) ratio of <60 % with incapacitating oedema should be treated with diuretics [140]. An important point to consider when obtaining urine for electrolyte analysis is that children should be on normal salt and water intake [138]. Additionally, diuretics, angiotensin-converting enzyme inhibitors (ACE-I) and angiotensin receptor blockers (ARB) may interfere with urine indices and hence should not be used before the urine indices have been tested or, alternatively, diuretics should be discontinued for at least 8 h. Kapur et al. presented data showing that diuretic therapy alone is safe and effective for severe oedema in overfilled children with INS, while underfilled patients may require both albumin and diuretics [137]. Diuretic use in underfilled patients (if used) requires diligent monitoring of the renal and systemic haemodynamic status to ensure that intravascular volume depletion is not exacerbated. Lower dose therapy is indicated initially. Overfilled patients may benefit from more aggressive diuretic therapy [141]. In mildly oedematous patients with a normal eGFR, many clinicians favour an oral thiazide diuretic as first-line therapy [141]. With severe oedema, intravenous loop diuretics may be more effective [142].

Loop diuretics block the sodium chloride co-transporter in the thick ascending limb of the loop of Henle [143]. Resistance to loop diuretics can occur and may partly be due to diuretic-induced negative sodium balance activating ‘sodium-retaining’ homeostatic mechanisms (e.g. sympathetic nervous system, renin–angiotensin–aldosterone system), thus functionally antagonising the intended diuretic effect. Concurrent use of a thiazide or amiloride may help under these circumstances [141]. However, poor drug compliance and dietary salt intake should first be excluded as apparent causes of loop diuretic-resistance. A trial of intravenous furosemide may also overcome apparent loop diuretic-resistance as it circumvents the poor bioavailability associated with an oedematous bowel wall.

Loop diuretics have a short duration of action (typically 6 h) and must be administered at least twice daily. Furosemide is a very commonly used loop diuretic, and one study in children with INS showed that 1 mg/kg of intravenous furosemide was twofold more effective than 2 mg/kg of oral furosemide [142]. If combination ‘loop and thiazide’ or ‘loop and thiazide-like (e.g. metolazone)’ diuretic therapy is considered necessary, careful monitoring to avoid hypokalaemia and alkalosis is required. The addition of amiloride or the mineralocorticoid receptor antagonist spironolactone to loop diuretic therapy can minimise hypokalaemia, although the absolute diuretic effect of these drugs is debatable [144, 145].

Failure of diuretic therapy in the oedematous child with INS may necessitate administration of intravenous loop diuretic therapy in conjunction with 20–25 % albumin solution. However, this treatment modality is not without risks: Haws et al. showed that although albumin and diuretic therapy results in fluid removal and weight loss in children with INS, the effect is transient unless remission of proteinuria occurs and it can be associated with serious adverse effects such as respiratory failure and congestive heart failure [146]. Reid et al. described three children with INS who required intensive care for life-threatening fluid overload and pulmonary oedema after receiving an excessive dose or a too rapid infusion of 20 % albumin solution, leading these authors to recommend 20 % albumin at a dose of 1 g/kg ideal body weight be given over 4 hours when warranted [147]. Albumin infusions can also be associated with allergic reactions, including anaphylaxis [148].

Careful assessment of a patient’s intravascular volume status is vital before commencing an albumin infusion. When an albumin infusion is required, fluid volumes should be calculated based on an estimated ‘dry’ weight rather than an oedematous weight. Albumin solution is likely to be more beneficial in children with INS who are underfilled in association with severe hypoalbuminaemia (<20 g/L). In contrast, the risk–benefit ratio is likely to be unfavourable in the child with INS who is oliguric or who has significantly reduced eGFR (due to the risk of pulmonary oedema); or in the child who is overfilled, as this may exacerbate the hypervolaemia and contribute to worsening systemic hypertension and pulmonary oedema [141].

**Fig. 1:**
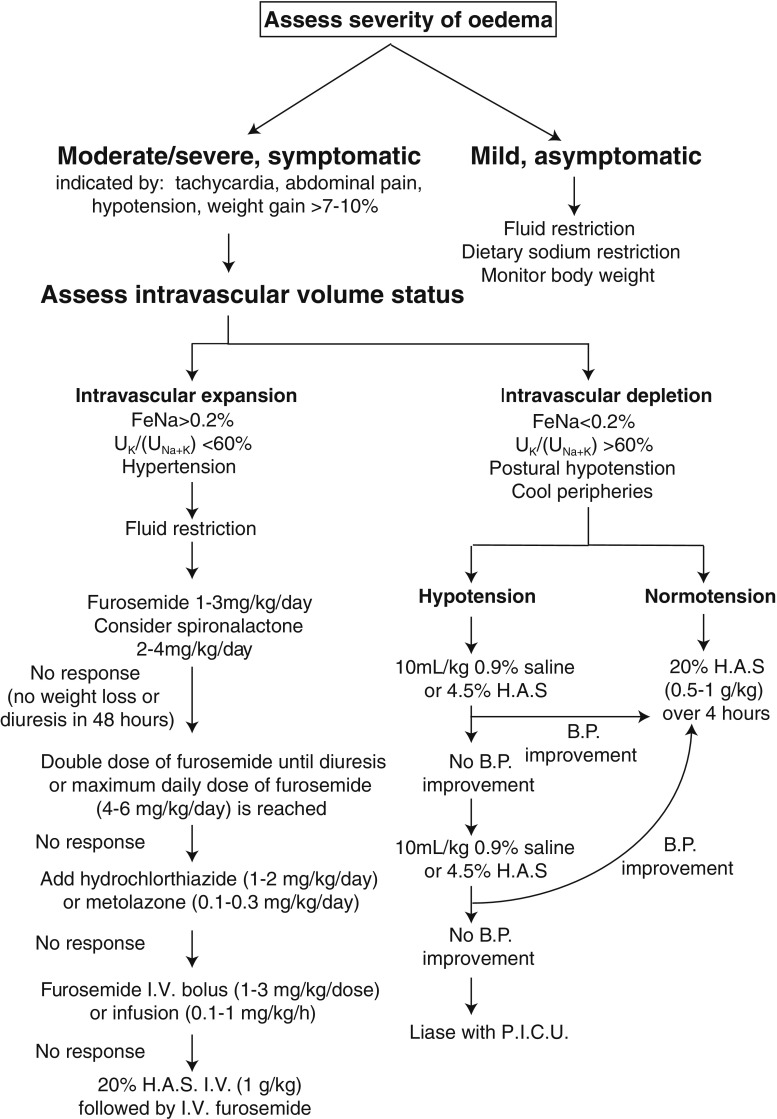
Modified Indian Academy of Paediatrics guidelines for the treatment of oedema in INS. Adapted from [19], used with permission from *Indian Pediatrics*. Patients should only receive diuretic therapy or albumin infusions under close clinical supervision. Fluid volumes should be calculated on an estimated ‘dry’ weight rather than an oedematous weight. Electrolyte levels should be monitored in all patients receiving diuretics, and potassium supplements/spironolactone started when necessary. *FeNa* Fractional excretion of sodium, *U*_*K*_/(*U*_*Na+K*_) quotient of urine potassium and urine sodium plus potassium, *I.V.* intravenous, *B.P.* blood pressure, *H.A.S.* human albumin solution, *P.I.C.U* paediatric intensive care unit

Some data have implicated stimulation of arginine vasopressin (AVP) from the pituitary gland as an important factor in oedema-formation in a rodent model of NS and adult NS patients [149, 150]. A case report documents the effective use of a selective oral vasopressin V2 receptor antagonist (tolvaptan) to treat a girl with NS-associated oedema, refractory to treatment with diuretics and albumin solution [127]. Several studies suggest that aberrant filtration of the urinary serine protease precursor plasminogen during periods of proteinuria, followed by conversion to its active form, plasmin, by renal tubular urokinase-type plasminogen activator causes activation of the epithelial sodium channel (ENaC) in the collecting duct [151, 152]. This observation may explain the inappropriate sodium retention often associated with INS and identifies urine serine protease inhibitors as another potential therapeutic target for treatment of oedema in INS. Further data on the use of these agents are required. The current IAP guidance on the treatment of oedema in INS is summarised in Fig. 1.

## Dyslipidaemia

Dyslipidaemia is a common feature of INS. Zilleruelo et al. studied 59 children with INS and found that serum total cholesterol and triglyceride levels were greater than or equal to the 95th percentile for age and sex in all children with minimal change NS (MCNS) in relapse and in children with non-MCNS with persistent nephrotic range proteinuria. Significantly decreased levels of the cardio-protective high-density lipoprotein (HDL) and persistent proteinuria were found in children with non-MCNS [153]. The pathophysiology is incompletely understood but may include increased hepatic 3-hydroxy-3-methylglutaryl-coenzyme A (HMG-CoA) reductase and acyl-coenzyme A-cholesterol acyltransferase activities or reduced lipoprotein lipase activity [154–156]. A role for the podocyte-secreted INS-triggering circulating factor angiopoietin-like 4 has also been suggested [157].

In children with INS, dyslipidaemia rapidly normalises following the disappearance of proteinuria [158]. It is unlikely that intermittent dyslipidaemia in childhood INS has any long-term consequences, although there is some anecdotal evidence of myocardial infarction and documented atherosclerosis in children with INS [159, 160]. As children with INS sometimes have several cardiovascular disease (CVD) risk factors in addition to dyslipidaemia (e.g. hypertension, Gc-induced obesity, insulin resistance), the potential risk of dyslipidaemia cannot be discounted on current evidence. INS in adults is associated with atherosclerosis and an increased risk for CVD [161]. However, in a study of 62 adults who had INS as children, the patients were found not to be increased risk for CVD mortality or morbidity compared to the general population [162].

Treatment with HMG-CoA reductase inhibitors (e.g. simvastatin, atorvastatin) has demonstrated a beneficial effect on dyslipidaemia in adult INS patients, but no data are available regarding the effect on long-term CVD health outcomes for this population [163, 164]. A meta-analysis of HMG-CoA reductase inhibitor use in the paediatric familial hypercholesterolaemia population has demonstrated short-term safety and efficacy [165]. HMG-CoA reductase inhibition blocks intracellular cholesterol synthesis, and the effects this has on cell growth, cell membrane and hormone synthesis are unknown. This is of particular concern for the pre-pubertal child. In rats, simvastatin and lovastatin were found to cause growth retardation and severe myopathy, although pravastatin did not show these effects [166]. RCTs involving HMG-CoA reductase inhibitors in the childhood INS population are lacking. When lipid-lowering agents are being considered in pre-pubertal children with INS, fibrate therapy may be a safer option than the use of HMG-CoA reductase inhibitors [158].

Dietary modification has been proposed as a potential treatment option to reduce the risk of CVD in adult NS patients. A vegan, low-protein diet [167] and vegetarian, low-protein diet [168] in adult INS patients was found to reduce serum cholesterol, but had no effect on triglyceride levels. Interestingly, one study found that treatment of 11 initially SRNS children with Gc therapy and low-density lipoprotein apheresis resulted in complete remission in five children and partial remission in two children [169]. The AAP recommends a low-fat diet in children with INS, as well as consideration of low-density lipoprotein cholesterol-lowering drug therapy, when fasting low-density lipoprotein cholesterol levels are persistently between >160 and 190 mg/dL (4.1– 4.9 mmol/L) [9].

## Obesity and growth

Two major side-effects of long-term, high-dose prednisolone treatment are growth impairment [170] and obesity [171]. A study of 80 children with SSNS examined 5–24 years after the initial diagnosis found that total Gc dose only correlated weakly with height standard deviation score (HSDS); there was also no correlation detected when post-pubertal children were studied separately [172]. Saha et al. studied 21 prepubertal children with steroid-dependent NS (SDNS) or frequently-relapsing NS before and during repeated oral Gc therapy and found that these children grew normally for their age before the onset of disease and that growth remained after disease onset despite Gc therapy [173]. Emma et al. conducted a study involving 42 children with SDNS and 14 children with frequently-relapsing NS and examined longitudinal height measurements over a mean period of 11.7 years. These authors found that during the pre-pubertal period, children lost 0.49 HSDS, although partial catch-up growth occurred after Gc withdrawal. The only statistically significant predictors of long-term outcome were mean duration of Gc therapy and average cumulative dose of Gc therapy [174]. A study of 29 boys and 12 girls with SSNS found that HSDS worsened significantly with chronological age for both boys and girls and that there was a significant negative correlation between the change in HSDS and duration of Gc treatment in boys, but not girls [175]. Berns et al. observed 60 children with INS for a minimum of 10 years from disease onset. Those children treated with Gc alone were −0.93 SD below the mean for height at last follow-up; in contrast, treatment with the Gc-sparing agent cyclophosphamide was associated with an increase in HSDS from −0.84 to −0.28 [176]. A similar observation was made in a study measuring the growth velocity of 12 children prior to and following treatment with either cyclophosphamide or chlorambucil, and alternate-day Gc therapy. The growth rate before the introduction of the alkylating agents was 4.3 cm/year, but this increased to 8.7 cm/year after therapy [177]. Hung et al. also found Gc therapy impaired linear growth in a dose-dependent manner but that combined administration of cyclophosphamide or chlorambucil reduced this effect [178].

A 2010 study has given important insight into the effects of prednisolone treatment specific to children with INS. Simmonds et al. studied the growth of 41 children with SDNS over a mean follow-up period of 4.2 years [179]. Overall, prednisolone treatment up to cumulative mean doses of prednisolone of 0.75 mg/kg/day was shown to not adversely affect HSDS. In those taking cumulative mean doses of >0.75 mg/kg/day, some decline in height SDS was seen during periods of higher steroid use, but periods on lower steroid doses allowed for adequate catch-up growth.

Evidence showing that steroid-sparing strategies may reduce obesity in children with INS comes from a study which demonstrated that following a reduction in prednisolone dose, obesity persisted in only two out of 13 initially obese children [50]. It should also be noted that no increased obesity rates [measured by body mass index (BMI)] were observed in adult patients who had experienced INS as children, and no correlation was found between weight in adulthood and cumulative Gc dose [11]. There is a paucity of data on the role of exercise in INS, but French guidance recommends that regular exercise should be undertaken. Monitoring of linear growth and BMI for children with INS is mandatory, and appropriate dietary counselling should be offered [9].

## Fracture risk

Children with NS may be at risk from steroid-induced osteoporosis and osteomalacia due to decreased 25-hydroxy-vitamin D levels secondary to urinary loss of vitamin D binding protein, potentially placing them at an increased risk of bone fractures [180, 181].

Gulati et al. prospectively studied 100 children with NS and found that 22 % had osteoporosis [quantitatively expressed as a bone mineral density (BMD) value evaluated by dual-energy X-linked absorptiometry (DEXA) of the lumbar spine] [182]. Additionally, a significant correlation was found between a lower BMD score and a greater cumulative steroid dose. Hegarty et al. also showed a significant reduction in forearm trabecular BMD in adults who suffered childhood INS [183]. In contrast, Leonard et al. examined 60 children with INS and 195 controls and found no deficits in spine or whole body bone mineral content [184]. It has not been shown that reduced BMD in children with INS results in an increased fracture rate. However, adults taking a prednisolone dose of ≥7.5 mg/day for either treatment of asthma or rheumatoid arthritis were found to have a twofold higher risk of experiencing a fracture [185].

A prospective study examined 100 children with INS over a mean of 1.5 years with serial DEXA imaging. Children who received supplemental calcium and vitamin D_3_ from study onset showed improved BMD scores compared to those not receiving supplementation [186]. The IAP recommends that children with INS receiving prolonged Gc therapy (>3 months) should receive daily supplements of oral calcium (250–500 mg) and vitamin D (125–250 IU) [19]. However, further trials are necessary to assess the role of calcium and vitamin D supplementation and to determine whether this therapy affects fracture risk in children with NS.

## Thyroid disease

Clinicians should be aware of thyroid disease in INS. Children with INS may experience low T4 and T3 levels secondary to urinary loss of thyroxine-binding globulin. However, serum levels of free throxine (FT4) and thyroid-stimulating hormone (TSH) are usually normal, and these children are considered to be euthyroid [187, 188]. Children with SRNS seem to have a higher risk of developing hypothyroidism than children with SSNS [188]. Children with INS have a state of mild/subclinical hypothyroidism during proteinuria although they are clinically euthyroid. This temporary state improves with remission and needs no treatment [189]. Ito et al. recommend that serum thyroid hormone concentrations be measured in children with INS and severe proteinuria persisting for >3 weeks despite Gc therapy and that thyroid replacement therapy, in addition to Gc, be provided to children with INS and evidence of moderate or severe hypothyroidism [190].

## Hypertension

Cardiovascular disease is the leading cause of death in the adult dialysis and renal transplant population [191]. The paediatric nephrologist should be alert to CVD risk factors in children with chronic renal disease and attempt to minimise these risks as early as possible. Hypertension is a major CVD risk factor, and a study of 57 children with INS found a prevalence of hypertension (defined as blood pressure >95th percentile for age) of 19 % [192]. The AAP recommend a low-salt diet, exercise and weight reduction if obesity is present. If blood pressure exceeds the 90th percentile of normal, anti-hypertensive pharmacological management in the form of ACE-I or ARB should be initiated.

## Persistent nephrosis

For children with SRNS, blockade of the renin–angiotensin system with either an ACE-I or angiotensin II receptor blocker ARB is recommended [9, 14]. These agents should also be considered in SDNS or frequently-relapsing NS. Two RCTs using the ACE-I enalapril and fosinopril have demonstrated a reduction in proteinuria in children with SRNS [193, 194]. In children with renal failure due to INS refractory to pharmacological management, bilateral nephrectomy by renal ablation or renal artery embolisation may be performed [195]. This procedure is also important in the pre-transplant setting to reduce thrombotic risks of the nephrotic state at renal transplantation.

## Conclusion

Children with INS generally have a favourable long-term outcome. A challenge for clinicians is to treat the immediate problems of proteinuria, altered fluid balance and infection, while minimising the long-term risk to health. A summary of major supportive therapies for INS is given in Table 2.

**Table 2:** Summary of treatment strategies in different phases of idiopathic nephrotic syndrome

Treatment strategies	Nephrotic state	Remission under immunosuppressive therapy	Remission after discontinuation of immunosuppressive therapy
Prophylactic antibiotics	✘	✘	✘
Pneumococcal vaccine	✘	✘ (ideally)	✓
Influenza vaccine	✘	✘	✓
Varicella vaccine	✘	✘	✓
Thromboprophylaxis	✘	✘	✘
Consideration of fluid restriction/diuretics/ albumin infusions	✓	✘	✘

NS is a complex disorder and more research needs to be performed to ensure informed management decisions are made. Infection should be treated promptly with broad-spectrum antibiotics in the nephrotic child. However, the role of antibiotic prophylaxis is still unclear, and vital data on the efficacy or otherwise of the pneumococcal vaccine in children with INS are lacking. Similarly, clinicians are aware of the potential devastating effects of TED in INS, but whether primary prophylaxis is warranted (for all children with INS or for a specific subgroup) is unknown. Further work is also needed to minimise the long-term CVD risk factor of dyslipidaemia in the INS population. Additionally, novel management strategies, such as modifying sialylation of the circulating glycoprotein angiopoietin-like 4, have shown promising results in rodent models of INS, and studies translating this to human disease are eagerly awaited [196, 197]. Substantial progress has been made in our ability to manage INS since the introduction of glucocorticoids and antibiotics in the second half of the last century, but continued progress through clinical trials is needed.
